# Clinical outcomes and the impact of treatment modalities in children with carbapenem-resistant Enterobacteriaceae bloodstream infections: a retrospective cohort study from a tertiary university hospital

**DOI:** 10.1093/jac/dkae387

**Published:** 2024-10-30

**Authors:** Gulhadiye Avcu, Ece Erci, Nimet Melis Bilen, Irem Ersayoglu, Gulcihan Ozek, Ulgen Celtik, Demet Terek, Feriha Cilli, Zumrut Sahbudak Bal

**Affiliations:** Faculty of Medicine, Department of Pediatrics, Division of Pediatric Infectious Diseases, Ege University, Izmir, Turkey; Faculty of Medicine, Department of Pediatrics, Division of Pediatric Infectious Diseases, Ege University, Izmir, Turkey; Faculty of Medicine, Department of Pediatrics, Division of Pediatric Infectious Diseases, Ege University, Izmir, Turkey; Faculty of Medicine, Department of Pediatrics, Division of Pediatric Intensive Care, Ege University, Izmir, Turkey; Faculty of Medicine, Department of Pediatrics, Division of Pediatric Hematology and Oncology, Ege University, Izmir, Turkey; Faculty of Medicine, Department of Pediatric Surgery, Ege University, Izmir, Turkey; Faculty of Medicine, Department of Neonatology, Ege University, Izmir, Turkey; Faculty of Medicine, Department of Medical Microbiology, Ege University, Izmir, Turkey; Faculty of Medicine, Department of Pediatrics, Division of Pediatric Infectious Diseases, Ege University, Izmir, Turkey

## Abstract

**Background:**

The increasing prevalence of carbapenem-resistant Enterobacteriaceae (CRE) infections among children represents a significant global concern, leading to elevated mortality rates. The aim of this study was to evaluate the risk factors, outcomes, 30-day mortality rates and contributing factors in children with CRE bloodstream infections (CRE-BSIs).

**Methods:**

Data regarding demographic characteristics, treatment approaches and outcomes of hospitalized children aged 0–18 years diagnosed with CRE-BSIs between January 2018 and December 2022 were extracted from medical records. Mortality within 30 days of diagnosis and the predictive factors were analysed.

**Results:**

A total of 114 children, with a median age of 11 months (range: 6–69.5), were included. All cases of CRE-BSIs were either healthcare associated or hospital acquired and presented with at least one underlying comorbidity. A previous history of CRE colonization or infection rate was 48.2% (55/114). *Klebsiella pneumoniae* 87.7% (100/114) was the most frequently isolated microorganism, with a 30-day mortality rate of 14% (16/114). Multivariate analysis identified paediatric intensive care unit admission, invasive mechanical ventilation, inotropic support and thrombocytopenia due to CRE-BSIs as the most discriminative predictors for 30-day mortality (*P* < 0.001). Central venous catheter (CVC) removal was associated with a reduced mortality rate (*P* = 0.012). High-dose prolonged infusion of MEM-based or polymyxin-based antibiotic combinations did not impact survival. Lower MEM MIC values were associated with improved survival.

**Conclusions:**

The mortality rate of CRE-BSI is notably high in childhood. The use of antibiotic combination strategies did not demonstrate a significant impact on 30-day survival; however, the removal of CVCs was found to lower mortality rates.

## Introduction

Infections with MDR Gram-negative bacilli, especially those resistant to carbapenems, are increasing worldwide. Among these, carbapenem-resistant Enterobacteriaceae (CRE) bloodstream infections (BSIs) are becoming increasingly problematic, affecting both adults and children and leading to substantial morbidity and mortality.^1–3^ Recent studies have highlighted concerning outcomes, with mortality rates reaching up to 52% in paediatric patients diagnosed with CRE-BSIs.^4–7^

Most episodes of CRE-BSIs reported in the literature are either hospital acquired or healthcare associated.^8,9^ Established risk factors for CRE-BSIs include underlying comorbidities such as immunosuppression, malignancy, intensive chemotherapy, severe neutropenia, prolonged hospitalization, prior central venous catheter (CVC) or mechanical ventilation (MV) use and exposure to broad-spectrum antibiotics.^10–12^ Management difficulties contribute significantly to mortality, including challenges in carbapenemase detection, limited access to appropriate targeted antibiotics in developing countries, age restrictions for certain antibiotics, insufficient availability of newly developed drugs, lack of comprehensive data on treatment outcomes and constraints in performing pharmacokinetic tests in children. Therefore, close monitoring of local surveillance data regarding colonization and infection, identification of risk factors and implementation of preventive measures are crucial for reducing mortality rates. As data on CRE-BSIs in children are limited, this study aims to evaluate risk factors, mortality rates and treatment approaches to enhance outcomes in paediatric patients.

## Materials and methods

### Study population and design

This single-centre retrospective observational study was conducted at Ege University Children's Hospital, a tertiary-level university hospital. It focused on hospitalized children aged 0–18 years diagnosed with CRE-BSIs between January 2018 and December 2022. The patients were retrospectively identified through the medical records and those who gave informed consent were included in the study. Two paediatric infectious disease specialists reviewed the medical records, and a standardized form was used to collect demographic data such as age, gender and comorbidities, as well as predisposing risk factors for CRE-BSIs, including invasive procedures (such as CVCs, MV, gastrostomy and tracheostomy), surgical interventions, total parenteral nutrition (TPN) and immunosuppression (primary/secondary). Additionally, parameters such as length of hospital stay, admission to paediatric intensive care unit (PICU) and medical management of CRE-BSIs (including empirical and targeted therapy, duration of treatment and whether monotherapy or combination therapy was used) were also evaluated.

Laboratory test results including complete blood count, C-reactive protein (CRP), procalcitonin, erythrocyte sedimentation rate and biochemical parameters such as urea, creatinine, AST, ALT, amylase, total protein, total and direct bilirubin levels, glucose, electrolytes, fibrinogen and coagulation parameters (prothrombin time, activated partial thromboplastin time and international normalized ratio) were obtained from patients’ medical records. Additionally, culture and antibiotic susceptibility profile were retrieved.

The inclusion criteria were as follows: (i) patients aged 0–18 years old (neonates were included), (ii) hospitalized between January 2018 and December 2022, (iii) diagnosed with CRE-BSIs based on blood cultures examinations and (iv) who agreed to participate (gave informed consent). If patients had >1 CRE-BSI episode, only the first episode was included and polymicrobial bacteraemia episodes were excluded in the study.

Central line-associated BSI (CLABSI) was defined according to the 2009 criteria established by IDSA.^13^

Hospital-acquired infections were defined by a positive blood culture obtained from patients who had been hospitalized for 48 h or longer. Healthcare-associated BSI was defined by a positive blood culture obtained from a patient with at least one of the four elements: (i) parenteral treatment within 30 days, (ii) outpatient chemotherapy or haemodialysis within 30 days, (iii) hospitalization for ≥2 days in the preceding 90 days and (4) nursing home residence.^14,15^

Day 1 for each participant was defined as the day when the initial positive blood culture was obtained, and all patients were followed for a period of 30 days after the first positive blood culture detection.

The outcomes and mortality of the patients within 30 days after the positive blood culture of CRE without a negative culture were reported.

### Microbiologic methods

Only non-duplicate isolates considered to be the causative pathogens of infection from paediatric patients were included in the study. All the strains were identified to the species level with MALDI-TOF MS (BioMérieux^®^). The antibiotic susceptibility testing of isolates was performed with the Vitek 2 automated system (bioMérieux, Marcy l'Etoile, France) and the gradient test.

### Statistical analysis

The SPSS statistics software was used to perform the statistical analysis (version 25 for Windows, IBM, Armonk, NY, USA). Mean, SD or medians (IQR) were used for continuous data, and percentages were used for categorical variables. The univariate analyses to identify variables associated with mortality were investigated using *χ*^2^, Fisher’s exact, student’s *t*- and Mann–Whitney *U*-tests, where appropriate. A *P* value of 0.05 was used to determine the statistical significance of differences and correlations. Predisposing factors with *P* value of <0.05 in univariate analysis were re-evaluated by multivariate analysis to determine independent predictors of mortality. Hosmer–Lemeshow's goodness-of-fit statistics was used to assess model fit. A 5% Type-I error level was used to infer statistical significance. The survival analysis used the Kaplan–Meier analysis, and a ‘log-rank test’ was performed to compare survival functions.

### Ethical approval

The Research Ethics Committee of Ege University Faculty of Medicine and the Ministry of Health approved the study (ethical decision no: 23-7T/32). This study was carried out in accordance with the Helsinki Declaration. The written consent of the children's parents was obtained.

## Results

### Patient characteristics

**Figure 1.**
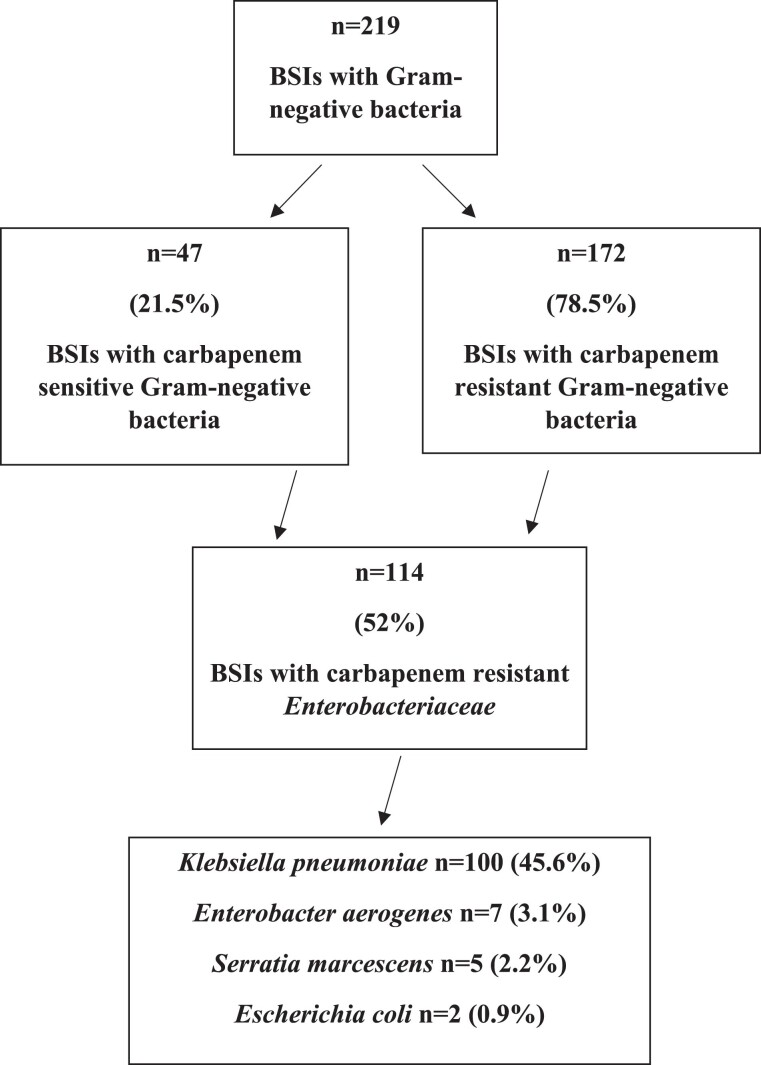
Selection of patients with CRE-BSIs.

There were 219 BSI episodes with Gram-negative bacteria among hospitalized children between January 2018 and December 2022. 78.5% (172/219) of these were caused by carbapenem-resistant Gram-negative bacteria, and 52% (114/219) were CRE (Figure 1). The median age was 11 months (6–69.5), and 66.6% (76/114) were male. All CRE-BSIs were either healthcare associated or hospital acquired. Within the last 12 months, there was a previous CRE colonization or infection rate of 48.2% (55/114).

All patients included in the study exhibited at least one underlying comorbidity. Specifically, 38.5% (44/114) had gastrointestinal diseases, and 35.9% (41/114) patients were immunosuppressed. The patient characteristics are detailed in Table 1.

**Table 1.** Characteristics of the hospitalized children with CRE-BSIs

	*n* (%)
Demographics of the patients (*n* = 114)	
Age (median, range)	11 months (6–69.5)
<3 months	24 (21.1)
3–36 months	55 (48.2)
>36 months	35 (30.7)
Sex, male	76 (66.6)
Comorbidities	114 (100)
GI disease	44 (38.5)
Short bowel	26 (22.8)
GI tract malformations	15 (13.1)
Liver failure	3 (2.6)
Immunosuppression	41 (35.9)
Haematological–oncological malignancy	16 (14)
Liver transplantation	5 (4.38)
Bone marrow transplantation	11 (9.6)
Primary immunodeficiency	9 (7.8)
Cardiopathy	10 (8.7)
Prematurity	8 (7.0)
Neurometabolic disorders	7(6.1)
Other chronic diseases	7(6.1)
Total length of hospital stay (day), median (range)	79.5 (51.5–260.7)
Length of hospital stay before infection	30 (13–107.2)
Length of hospital stay after infection	43.5 (23–75.5)
Infection site	
Peripheral blood	39 (34.2)
CLABSI	75 (65.8)
Microorganisms reported	
*Klebsiella pneumoniae*	100 (87.7)
*Enterobacter cloacae*	7 (6.1)
*Serratia marcescens*	5 (4.4)
*Escherichia coli*	2 (1.7)
Meropenem susceptibility	50 (43.8)
MIC < 4–8 mg/L	6 (5.2)
MIC > 8 mg/L	44 (38.5)
MIC > 32 mg/L	17 (14.9)
Laboratory parameters (mean ± SD)	
WBC (µL)	9981 ± 9687
ANC (µL)	6592 ± 8070
PLT (10^3^/µL)	205 ± 183
CRP, mg/L	150 ± 643
PCT, µg/L	25.5 ± 74.2
30-day mortality	16(14)

ANC, absolute neutrophil count; BSI, bloodstream infection; CLABSI, central line-associated bloodstream infection; CRE, carbapenem-resistant Enterobacteriaceae; CRP, C-reactive protein; GI: gastrointestinal; PCT, procalcitonin; PLT, platelet count; WBC, white blood cell.

### Microbiological results

Of 114 patients with CRE-BSIs, 65.8% (75/114) patients had CLABSI and only peripheral blood culture yielded CRE in 34.2% (39/114) of the patients. The median time to obtain a negative culture was 5 (range; 3–9) days [it was 6 (3–10.5) for CLABSI patients] and catheter removal was achieved in 70.6% (53/75) of the CLABSIs. Among patients who developed CLABSIs and could not achieve negative blood cultures, eight patients with port catheters and four out of six patients with CVCs were unable to have their catheters removed. In the non-CLABSI group, two patients also could not obtain negative cultures. Septic shock caused death in both groups before achieving negative blood cultures.

*Klebsiella pneumoniae* (87.7%) was the most frequently isolated microorganism, followed by *Enterobacter cloacae* (6.1%), *Serratia marcescens* (4.4%) and *Escherichia coli* (1.7%). MEM susceptibility was available in 43.8% (50/114) of the patients, and 44 had a MIC value of >8 mg/L (Table 1).

### Risk factors for CRE bacteraemia

The potential risk factors of the patients for CRE-BSIs in the last 3 months were examined. Before the current CRE-BSI, 70.1% (80/114) of patients were hospitalized and 54.4% (62/114) were admitted to the PICU. 76.3% (87/114) of the patients had received a broad-spectrum antimicrobial, and 62.2% (71/114) had carbapenem exposure. Most children had CVC (74.6%, 85/114), received TPN (74.6%, 85/114) and underwent surgical intervention (52.6%, 60/114). The identified risk factors are summarized in Table 2.

**Table 2.** Predisposing factors for CRE-BSIs

Previous colonization or infection of CRE	55 (48.2%)
Previous hospitalization	80 (70.1)
Length of hospital stay before infection (previous 3 months) (day), median (range)	30 (13–107.2)
PICU admission(previous 3 months)	62 (54.4)
Length of stay at PICU (day), median (range)	4.5 (0–23.5)
Mechanical ventilation (previous 3 months)	43 (37.7)
Previous interventions (previous 3 months)	
Medications	
Any antimicrobials	87 (76.3)
Carbapenem exposure (last 3 months)	29 (25.4)
Ongoing carbapenem (lasting at least 48 h)	42 (36.8)
Corticosteroids	38 (33.3)
Immunosuppressants	56 (49.1)
Surgery	60 (52.6)
Total parenteral nutrition	85 (74.6)
Invasive procedures and devices	
Central venous catheterization	85 (74.6)
Central venous catheter	64 (56.1)
Port catheter	20 (17.5)
PICC line	1 (0.8)
Tracheostomy	19 (16.7)
Gastrostomy	13 (11.4)
Nasogastric tube	68 (59.6)
Urinary catheter	27 (23.7)

CRE, carbapenem-resistant Enterobacteriaceae; PICC, peripherally inserted central cathater; PICU, paediatric intensive care unit.

### Management

Out of the total patients, 60.5% (69/114) were monitored at PICU with 46.4% (53/114) previously hospitalized and 14% (16/114) newly admitted due to CRE-BSIs. Among them, 50.7% (35/69) of the patients required MV, and 39.1% (27/69) received inotropic support. The median duration of PICU stay during the CRE-BSI episode was 4 days (ranging from 1 to 28 days).

High-dose prolonged infusion of MEM (3 × 40 mg/kg/dose over 3 h) with a second active agent [aminoglycosides (AMK, 15 mg/kg/daily; GEN, 5–7.5 mg/kg/daily), quinolones (CIP, 20 mg/kg/daily) or other carbapenems (ETP, 30 mg/kg/daily and 1000 mg once daily in adolescents; IPM/cilastatin, 100 mg/kg/daily)] was one of the active treatments in patients with CRE-BSIs. Even though it was not always possible to perform CST susceptibility, CST (3–5 mg/kg/day) with a second active agent (aminoglycosides or quinolones) was another treatment modality. In case of treatment failure, salvage treatment included triple-quadruple antimicrobial combinations and the addition of TGC (2–4 mg/kg/daily and 2 × 50 mg daily in adolescents). The antimicrobial treatment duration for CRE-BSI was 14 days in patients with microbiological eradication.

At the time of the initial positive blood culture, 76.3% (87/114) of the patients were already receiving antimicrobial therapy, with 36.8% (42/114) specifically on MEM treatment. Treatment adjustments were made for 73.6% (84/114) of the patients, with 71.9% (82/114) receiving combination therapy. Among those receiving combination therapy, 35% (40/114) were treated with MEM-based regimens, while 37.7% (43/114) received CST-based combinations. CZA was used in 7% (8/114) of the patients. Transient renal failure occurred in 3.5% (4/114) of patients, which was associated with various combination therapies.

### Outcomes and mortality

The CRE-BSI-related 30-day mortality rate was 14% (16/114). Multivariate logistic regression analysis for predictors of mortality showed that previous PICU admission (OR: 0.110, 95% CI: 0.019–0.647, *P* = 0.01), PICU admission due to CRE-BSIs (OR: 14.143, 95% CI: 4.033–49.596, *P* < 0.01), MV (OR: 22.237, 95% CI: 4.608–107.314, *P* < 0.01), receiving inotropic support (OR: 4.842, 95% CI: 1.273–18.421, *P* < 0.01) and thrombocytopenia due to CRE-BSIs (OR: 4.841, 95% CI: 1.273–18.421, *P* = 0.02) increased the mortality. Despite that, removing the CVC in CLABSIs decreased the mortality rate (OR: 12.80, 95% CI: 1.741–94.137, *P* = 0.01) (Table 3).

**Table 3.** Multivariate analysis of risk factors for 30-day mortality in children with CRE-BSIs

Risk factor	*P* value	OR (95% CI)
Age at the diagnosis (<2 years)	0.51	0.667 (0.197–2.256)
previous CRE colonization	0.79	0.887 (0.356–2.210)
Previous exposure to immunosuppressants	0.64	1.761 (0.158–19.679)
Prolonged exposure to carbapenems (lasting longer than >14 days)	0.28	0.314 (0.380–2.619)
PICU admission before CRE-BSI	**0.01**	0.110 (0.019–0.647)
GI surgery	0.63	0.643 (0.106–3.901)
Meropenem MIC > 8 mg/L	0.24	0.313 (0.045–2.178)
Meropenem MIC > 32 mg/L	0.81	1.250 (0.197–7.942)
Colistin-based combination therapy	0.55	1.675 (0.302–9.273)
Extended infusion of high-dose meropenem-based combination therapy	0.41	0.565 (0.146–2.196)
Prolonged time to obtain a negative culture (lasting longer than >7 days)	0.27	2.256 (0.529–9.633)
Removal of the catheter	**0**.**01**	12.80 (1.741–94.137)
PICU admission due to CRE-BSI	**<0**.**01**	14.143 (4.033–49.596)
Mechanical ventilation	**<0**.**01**	22.237 (4.608–107.314)
Inotrope support	**<0**.**01**	4.842 (1.273–18.421)
Neutropenia	0.36	1.818 (0.505–6.541)
Thrombocytopenia	**0**.**02**	4.841 (1.273–18.421)
CRP (>50 mg/L)	0.284	0.557 (0.191–1.626)
PCT (>5 ng/mL)	0.476	2.217 (0.248–19.791)

Statistical significance of differences were defined *P* value of < 0.05. BSI, bloodstream infection; CRE, carbapenem-resistant Enterobacteriaceae; CRP, C-reactive protein; GI, gastrointestinal; PCT, procalcitonin; PICU, paediatric intensive care unit.

**Figure 2.**
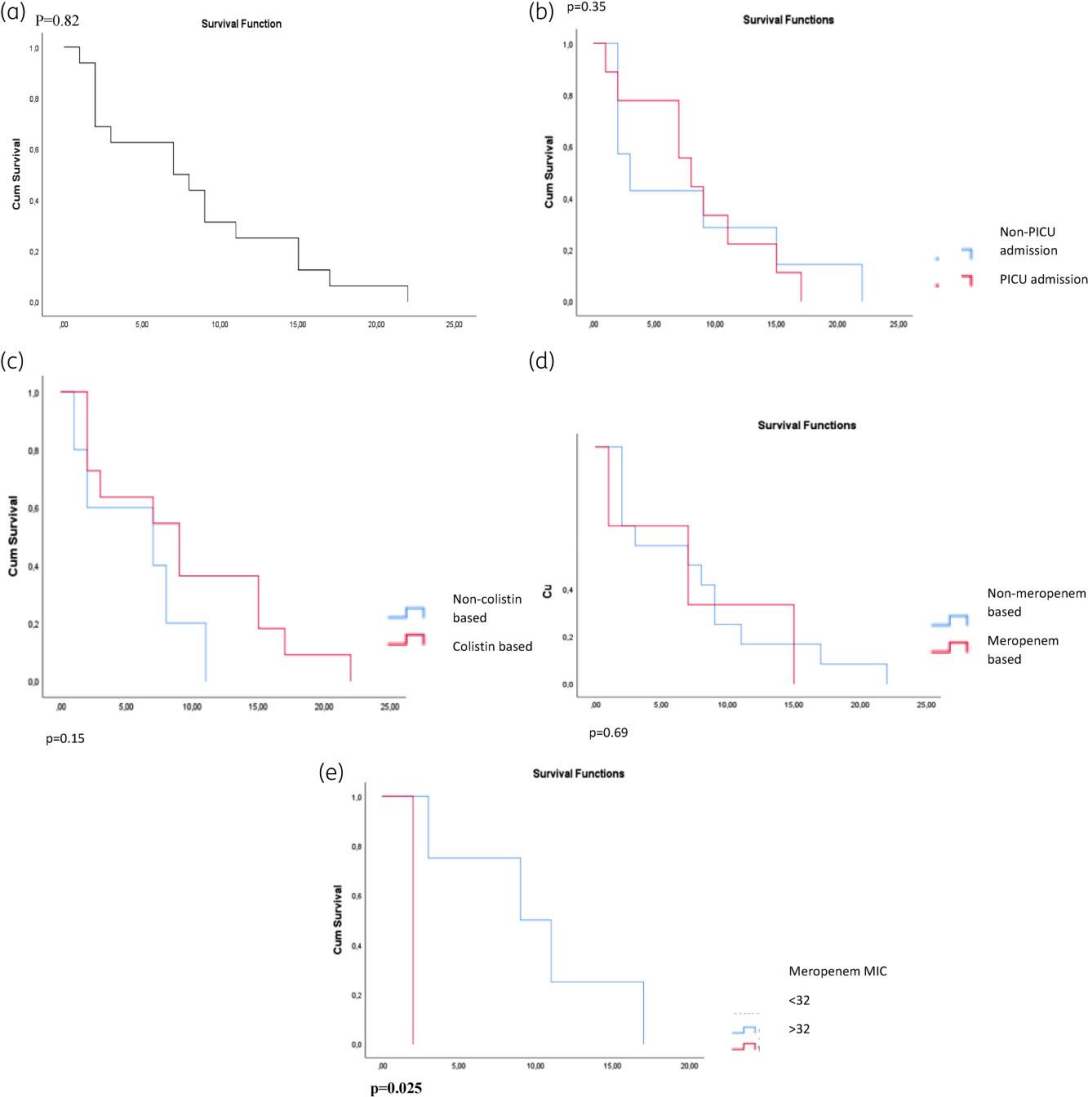
Kaplan–Meier curves of prognostic factors and survival at 30 days in children with CRE-BSIs. (a) total group patients; (b) PICU admission; (c) colistin-based combination therapy; (d) meropenem-based combination therapy; and (e) meropenem MIC value. This figure appears in colour in the online version of *JAC* and in black and white in the print version of *JAC*.

In our series, survival was unrelated to treatment modalities in management. No difference was detected between CST-based or MEM-based combination therapies in terms of survival outcomes. However, it was noted that higher MEM MIC values had a negative impact on survival (Figure 2).

## Discussion

This study presents findings from a 5-year single-centre investigation involving hospitalized children with CRE-BSIs, encompassing data from 114 patients. Each patient exhibited at least one comorbidity, with gastrointestinal disorders (38.5%), particularly short bowel syndrome and gastrointestinal tract malformations, and immunosuppression (35.9%)—either primary or secondary—being the most prevalent underlying conditions. Numerous prior articles have highlighted that the cohorts most susceptible to CRE-BSIs include individuals with haematological–oncological malignancies and those who are immunocompromised.^11,16^

All cases of CRE-BSIs in our study were associated with healthcare facilities or acquired during hospitalization, aligning with findings from similar paediatric studies.^8,9^ Prior antibiotic exposure (β-lactam/β-lactamase inhibitors, anti-pseudomonal PENs, extended-spectrum cephalosporins, carbapenems, glycopeptides and quinolones), immunosuppression, the presence of indwelling devices, prolonged stays in the intensive care unit, surgical interventions, solid organ transplantation and renal failure have been identified as risk factors for CRE infection.^8,16–18^

Our study revealed that previous colonization or infection with CRE, prior hospitalization and antibiotic exposure, invasive procedures and PICU admission were predisposing risk factors for CRE-BSIs, which were in line with previous reports showing that these factors increase the risk of acquiring CRE-BSIs.

*K. pneumoniae* (100/114) emerged as the predominant pathogen among our patient cohort, consistent with recent epidemiological findings where it ranks as the most frequently isolated pathogen in children with CRE infections.^5,11^ MEM susceptibility data were accessible for 43.9% of patients, revealing notably high MIC values (MIC > 8, 38.5%; MIC > 32, 14.9%).

The CRE-BSI-related 30-day mortality rate was 14% in the present study, which ranged from 11.5% to 41% among children with CRE-BSIs in the past series.^12,19,20^ Of the patients, 65.8% had CLABSI, and catheter removal was achieved in 70.6% of them. Our low mortality rate can be attributed to the prompt evaluation of those diagnosed with CRE-BSI by the paediatric infection team, rapid action in the decision to remove the catheter, which was statistically significantly associated with reduced mortality (*P* = 0.01, OR: 12.80, 95% CI: 1.741–94.137).

Predictors associated with mortality included previous PICU admission, admission due to CRE-BSI episode, receiving inotropic support and thrombocytopenia due to the episode in this cohort. High platelet levels are associated with reduced mortality rates in a recent study evaluating neonates and non-neonates with CR-*K. pneumoniae* BSIs.^12^ Severe sepsis and respiratory source, intensive care admission, intubation and administration of inotropes were reported as the mortality-associated factors in a large recent paediatric cohort, and the 30-day mortality rate has been reported as high as 52%.^5^ They reported that a MIC value of >8 mg/L for the isolate and failure to clear bacteraemia was associated with a statistical increase in mortality. Madney *et al*.^21^ reported that carbapenem resistance with a MIC > 8 mg/L was associated with a higher mortality rate in paediatric cancer patients suffering from CRE-BSIs. MIC values for the isolates have been suggested as a prognostic factor in CRE infections in some other studies.^22,23^ Besides, prolonged time to obtain a negative culture and high MIC values were not associated with mortality in this study.

High MEM MIC values (MIC > 32 mg/L) negatively affected the survival of our patients. In contrast, CST-based combination therapy and extended infusion of high-dose MEM-based combination therapy were not associated with improved survival. In contrast, Ara-Montojo *et al*.^24^ mentioned that a low MEM MIC was not related to improved survival. Septic shock on presentation, inadequate empirical antimicrobial therapy and delayed adequate active treatment more than 48 h were reported to be statistically significantly associated with poor survival.^25^

Like many developing countries, obtaining carbapenemase gene data was not feasible at our centre. Most new antimicrobials were reported to be effective in the treatment of CRE (MEM vaborbactam, IPM/cilastatin-relebactam, eravacycline, FDC, etc.). There are a limited number of antimicrobials that can be used in the management of these patients due to the lack of FDA approval, insufficient data on their use in the paediatric age group and access problems in developing countries. Access to targeted antimicrobials is also limited like CZA, which is a very good option, in developing countries due to the lack of availability to hospitals and restrictions to the use of the limited number of antimicrobials available to clinicians (cannot be used except for patients hospitalized in intensive care units, etc.).

Therefore, the impact of our treatment approaches, particularly combination therapies, on mortality and survival rates held significant importance in guiding our future clinical practices. Observational studies have indicated that combination therapy is often preferred in managing CRE infections. For instance, Nabarro *et al*.^5^ noted a high level of aminoglycoside resistance and highlighted lower mortality rates associated with the use of two or more effective drugs in combination. Similarly, an Italian Retrospective Multicenter Study found that combined therapy was significantly more common among infected patients with CREs (61.8%).^6^ Montagnani *et al*.^25^ reported reduced survival rates in patients treated with CST. In our study, combination therapy was administered to 71.9% of patients. However, neither CST-based combination therapy nor extended infusion of high-dose MEM-based combination therapy showed a significant effect on mortality rates.

This study has several limitations, including its retrospective design, which reflects a single-centre experience. Additionally, MIC values were unavailable for over half of the cases, and the study was unable to detect carbapenemase genes. Furthermore, the management often favoured combination therapy, with various antimicrobial combinations used; however, the lack of pharmacokinetic analysis (therapeutic blood levels) obscured which agent was more effective in achieving cure and improving survival rates.

Nevertheless, our study benefits from being conducted at a tertiary university hospital, enabling a multidisciplinary approach and management by a paediatric infectious diseases specialist based on appropriate indications. We were able to identify risk factors and mortality rates, providing valuable data to guide the adaptation of infection control programmes aimed at preventing CR infections among hospitalized paediatric patients.

In summary, this study highlights a notably elevated mortality rate among children with CRE-BSIs. The predictors of mortality align with previous findings, underscoring the criticality of demonstrating the life-saving impact of catheter removal. Furthermore, the study indicates that antibiotic combination strategies did not influence mortality within this cohort. Prospective studies focusing specifically on treatment approaches in childhood will provide crucial information for clinicians and help to reduce mortality rates, especially due to difficulties in their management and treatment failures.
